# Serum CYR61 Is Associated With Airway Inflammation and Is a Potential Biomarker for Severity in Chronic Obstructive Pulmonary Disease

**DOI:** 10.3389/fmed.2021.781596

**Published:** 2021-11-30

**Authors:** Zhu-Xia Tan, Lin Fu, Wen-Jing Wang, Ping Zhan, Hui Zhao, Hua Wang, De-Xiang Xu

**Affiliations:** ^1^Department of Toxicology, Anhui Medical University, Hefei, China; ^2^Second Affiliated Hospital, Anhui Medical University, Hefei, China

**Keywords:** CYR61, COPD, NF-κB, lung function, inflammatory cytokines

## Abstract

**Background:** Cysteine-rich 61 (CYR61) and inflammation was upregulated in the lungs of patients with chronic obstructive pulmonary disease (COPD). However, the association between CYR61 and inflammation was unclear in COPD patients. This study aimed to analyze the association of serum CYR61 with pulmonary inflammation and lung function indexes in COPD patients.

**Methods:** One hundred and fifty COPD patients and 150 control subjects were enrolled. Serum and pulmonary CYR61 was detected. Lung function indexes were evaluated in COPD patients.

**Results:** Serum CYR61 level was elevated and pulmonary CYR61 expression was upregulated in COPD patients. An increased CYR61 was associated with decreased pulmonary function indexes in COPD patients. Further analyses showed that nuclear factor-kappa B (NF-κB) p65-positive nuclei was elevated in the lungs of COPD patients with high level of CYR61. Accordingly, serum monocyte chemotactic protein (MCP)-1 and tumor necrosis factor α (TNF-α), two downstream inflammatory cytokines of NF-κB pathway, were increased in parallel with CYR61, among which serum MCP-1 and TNF-α were the highest in COPD patients with high level of CYR61. Moreover, a positive correlation, determined by multivariate regression that excluded the influence of age, gender and smoking, was observed between serum CYR61 and inflammatory cytokines in COPD patients.

**Conclusion:** These results provide evidence that an increased CYR61 is associated with pulmonary inflammation and COPD progression. Inflammatory cytokines may be the mediators between CYR61 elevation and COPD progression.

## Background

Chronic obstructive pulmonary disease (COPD) is a common respiratory disease, which is characterized by lung parenchyma damage and progressive decline in lung function (1–3). Cigarette smoking is a major risk factor for the occurrence and development of COPD (4). Chronic airway inflammation, accompanied by infiltration of numerous macrophages and lymphocytes, has been implicated in the progression of COPD (5–7). Accumulating data have demonstrated that chemokines, such as monocyte chemotactic protein (MCP)-1 and interleukin (IL)-8, are involved in the recruitment of inflammatory cells (8, 9). The activation of nuclear factor-kappa B (NF-κB) may play important roles in regulating cigarette smoke-evoked inflammatory chemokines (10–13).

Cysteine-rich 61 (CYR61), also named as CCN1, is a member of CCN protein family (14). Numerous data have demonstrated that CYR61 takes part in the process of angiogenesis, embryonic development, and tissue repair (15–17). Recently, the role of CYR61 in pulmonary diseases is concerned (18). Several studies indicated that CYR61 was involved in the pathogenesis of acute lung injury and acute respiratory distress syndrome (19, 20). Moreover, CYR61 aggravated transforming growth factor (TGF)-β-induced SMAD3 activation and lung fibrosis (21). An early report showed that pulmonary CYR61 expression was upregulated in COPD patients (22). Nevertheless, the association between upregulated CYR61 and COPD progression remains unknown.

In the current study, we aimed to analyze the association among serum CYR61, pulmonary inflammation and lung function indexes in COPD patients. We showed that serum CYR61 was elevated and pulmonary CYR61 expression was upregulated in COPD patients. Moreover, an elevation of serum CYR61 was associated with lung function decline in COPD patients. Our results provide evidence that inflammatory chemokine MCP-1 is a mediator between an increased CYR61 and lung function decline in COPD patients.

## Methods

### Reagents and Chemicals

Antibodies against NF-κB p65 and CYR61 were purchased from Cell Signaling Technology (MA, USA). CYR61 ELISA kits were from Cusabio (TX, USA). MCP-1 ELISA kit was from Wuhan Colorful Gene Biological Technology (Hubei, China). Chemiluminescence (ECL) detection kits were from Advansta (CA, USA). All other regents and chemicals were from Sigma Chemical Co. (MO, USA) if not specifically noted.

### COPD Patients and Lung Specimen

All COPD patients who were first time diagnosed were randomly selected from Anhui COPD Cohort (AHCC) that was a hospital-based prospective cohort established by the Second Affiliated Hospital of Anhui Medical University. For the matched case-control study, 150 COPD patients were recruited from AHCC. Pulmonary function was tested in all recruited COPD patients based on standardized methods. COPD was confirmed on basis of the American Thoracic Society criteria and the Global Initiative for COPD (GOLD) criteria (23), which forced expiratory volume in 1 s (FEV1)/forced vital capacity (FVC) ratio was <70%. Total 150 sex- and age-matched control subjects were randomly collected from the physical examination center at the Second Affiliated Hospital of Anhui Medical University. To analyze the level of serum CYR61 and MCP-1, sera were collected from all COPD patients and controls. To measure pulmonary CYR61 and NF-κB, lung tissues were obtained from surgical operations between COPD patients and Controls. Lung specimens were collected from paracancerous tissue of lung cancer patients without other pulmonary disease were as controls at the Second Affiliated Hospital of Anhui Medical University. Each control subject was matched with one COPD patients in accord with age and gender (24). Finally, all 20 lung cancer patients without other pulmonary diseases and 20 COPD patients were enrolled. The current study was approved by the Ethics Committee of Anhui Medical University (2021030). All subjects have agreed and signed an informed consent.

### Immunochemistry (IHC)

All lung specimens were fixed in formalin and embedded in paraffin. Lung section was dewaxed and rehydrated according to a conventional standard method (25). To punch cell membrane and suppress endogenous peroxidase, lung section was immersed in PBS containing 0.5% Triton X-100 and 3% H_2_O_2_ for 45 min. Antigen retrieval was performed in boiled citrate solution. After blocked, lung section was incubated with either CYR61 or NF-κB p65 antibody (1:200) at 37°C incubator for 3.5 h. After washed with PBS for three times, conjunction with streptavidin-HRP complex was incubated for 2.5 h at room temperature. Immunolabelling was evaluated using DAB solution. nucleus was stained with hematoxylin in a dark room. Pulmonary CYR61- and p65- positive cells were calculated by two independent pathologists.

### Enzyme Linked Immunosorbent Assay

Serum concentrations of CYR61, monocyte chemoattractant protein 1 (MCP-1) and tumor necrosis factor α (TNF-α) were measured using enzyme linked immunosorbent assay (ELISA). CYR61 (CSB-E13884h) and MCP-1 (CSB-E04655h) ELISA kits were bought from Cusabio, Wuhan, China (https://www.cusabio.com/). TNF-α (JYM0110Hu) ELISA kits were obtained from Wuhan ColorfulGene Biological Technology Co (http://www.jymbio.com/product/286-cn.html). The detailed method referred to the reagent manual (26).

### Statistical Analysis

The quantitative variables were expressed as means and standard error of mean. The categorical variables were expressed with frequencies and percentages. All statistical analysis was conducted in SPSS 21.0. Independent sample unpaired *t*-test was used to evaluate the difference for continuous variables between two groups. The difference of continuous variables in three groups was determined through one-way ANOVA. Chi-square test was used to analyze the difference for count data. Pearson correlation analysis and linear regression analysis were used to evaluate the correlations among CYR61 and inflammatory cytokines. The association of serum CYR61 and hospital stays was accessed through logistical regression analysis. *P* < 0.05 was considered statistically significant.

## Results

### Demographic Data and Clinical Characteristics

The demographic data and clinical characteristics were analyzed. As shown in Table 1, 150 COPD patients and 150 controls were recruited in this study. No significant difference on mean ages was observed between two groups (72.83 ± 0.61 in COPD patients vs. 67.94 ± 0.94 in controls, *P* > 0.05). In addition, no significant difference on sex ratio was shown between two groups (Table 1). There were 121 (80.7%) cases with emphysema in COPD patients (Table 1). Interestingly, there was obvious difference of smokers between COPD patients and control subjects. Blood routine indexes were then analyzed. As expected, the counts of white blood cell (WBC), neutrophil, eosinophil, monocyte and basophil were elevated in COPD patients (Table 1). By contrast, lymphocyte count was reduced in COPD patients (Table 1). The results of FEV1(%), FEV1/FVC, FEV1 (L), FVC (L), PH, PCO2, PO2, RV%TLC-SB (%), and DLCO SB in COPD patients was presented in Table 1. Not only that, the demographic data and clinical characteristics were further compared in COPD patients with different grades. As shown in Supplementary Table 1, no difference of emphysema and blood gas indicators was observed in COPD patients with different grades. RV%TLC-SB was lower and DLCO SB was higher in grade 1-2 (G 1-2) COPD patients than those in grade 3 and grade 4 (G 3 and G 4) COPD cases (Supplementary Table 1).

**Table 1:** Demographic information and clinical characteristics.

**Variable**	**Control (*n* = 150)**	**COPD (*n* = 150)**	***P***
Age (years)	67.9 ± 0.94	72.8 ± 0.61	0.132
Female, *n* (%)	51 (34.0)	38 (25.3)	0.100
Emphysema	N.A.	121 (80.7)	N.A.
Smoking status			<0.001
Never-smoker, *n* (%)	55 (36.7)	8 (5.3)	
Former-smoker, *n* (%)	65 (43.3)	123 (82.0)	
Current-smoker, *n* (%)	30 (20.0)	19 (12.7)	
WBC (10^9^/L)	6.22 ± 0.121	7.45 ± 0.278	<0.01
Neutrophil (10^9^/L)	3.24 ± 0.077	5.40 ± 0.270	<0.01
Lymphocyte (10^9^/L)	1.83 ± 0.071	1.26 ± 0.047	<0.01
Eosinophil (10^9^/L)	0.12 ± 0.010	0.18 ± 0.024	0.001
Monocyte (10^9^/L)	0.41 ± 0.056	0.57 ± 0.033	<0.01
Basophil (10^9^/L)	0.02 ± 0.002	0.03 ± 0.003	0.009
FEV1 (%)	N.A.	52.02 ± 2.659	N.A.
FEV1/FVC (%)	N.A.	57.79 ± 1.537	N.A.
FEV1 (L)	N.A.	1.16 ± 0.066	N.A.
FVC (L)	N.A.	1.88 ± 0.064	N.A.
PH	N.A.	7.39 ± 0.007	N.A.
PCO2 (mmHg)	N.A.	51.99 ± 2.107	N.A.
PO2 (mmHg)	N.A.	70.88 ± 2.588	N.A.
RV%TLC-SB (%)	N.A.	54.59 ± 1.564	N.A.
DLCO SB (mmol/min/kPa)	N.A.	3.82 ± 0.259	N.A.

*WBC, White blood cell; FEV1, Forced expiratory volume in one second; FVC, Forced vital capacity; N.A., Not available*.

### An Increased CYR61 Is Associated With the Severity of COPD

**Figure 1:**
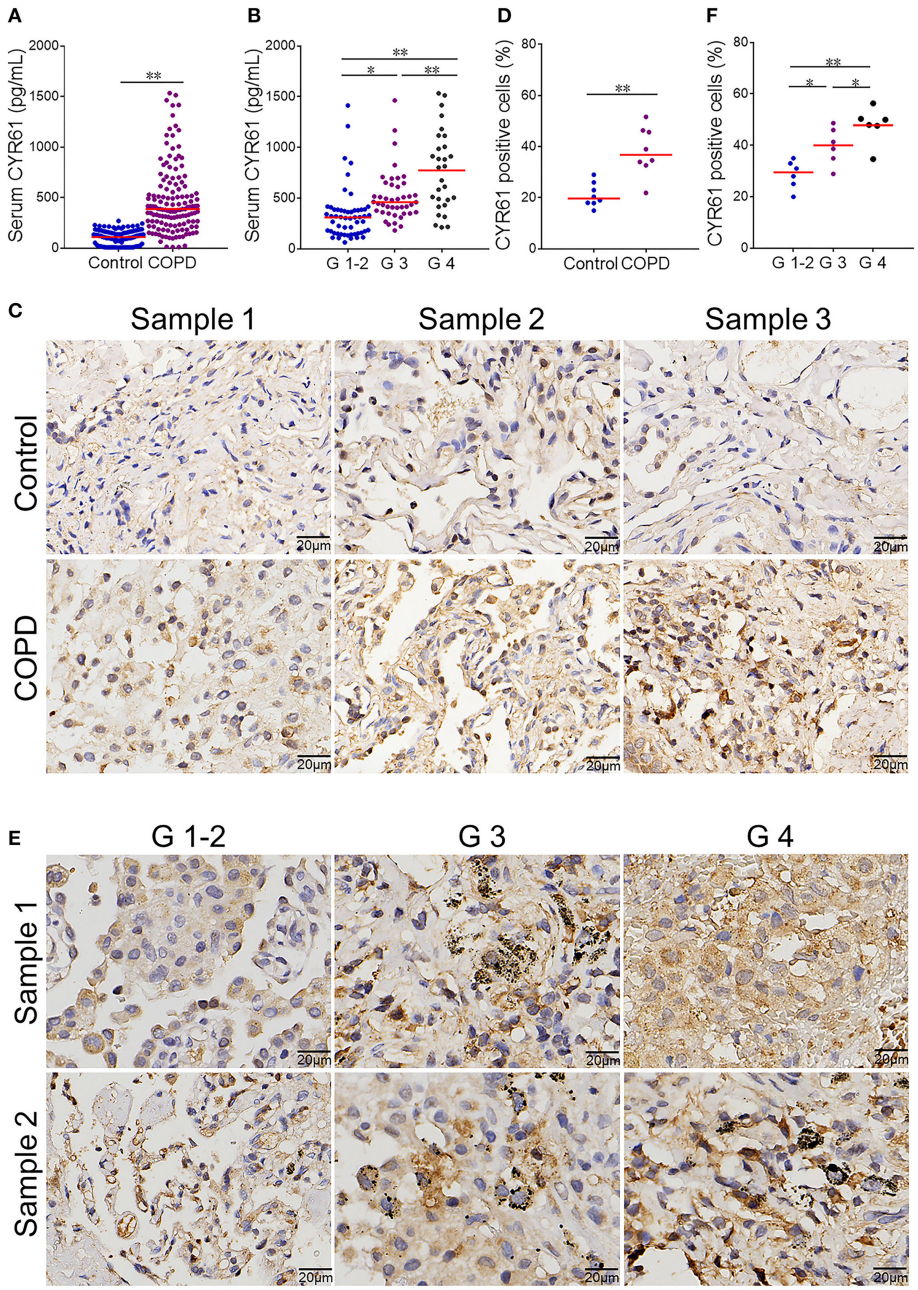
Serum CYR61 level and its association with severity of COPD. Sera were collected from COPD patients and controls. **(A,B)** Serum CYR61 level was detected using ELISA. **(A)** Serum CYR61 level was compared between COPD patients and controls. All data were represented as means ± S.E.M. (*N* = 150). **(B)** Serum CYR61 level was compared among different grades of COPD patients. All data were represented as means ± S.E.M. (*N* = 69 for G 1-2 patients, *N* = 48 for G 3 patients, *N* = 33 for G 4 patients). **(C,D)** Pulmonary CYR61 expression was compared between COPD patients and controls. **(C)** Three representative pictures. **(D)** Quantitative analysis of CYR61-positive cells in COPD patients and controls. **(E,F)** Pulmonary CYR61 was compared among different grades of COPD patients. **(E)** Three representative pictures: arrows indicate CYR61-positive cell; **(F)** Quantitative analysis of CYR61-positive cells in COPD patients with different grades. All data were represented as means ± S.E.M. (*N* = 6). *P < 0.05, **P < 0.01.

Serum CYR61 concentration was analyzed in COPD patients and controls. As shown in Figure 1A, serum CYR61 was elevated in COPD patients as compared with controls. Serum CYR61 was then compared among different grades of COPD patients. As shown in Figure 1B, serum CYR61 was gradually elevated in parallel with the grades of COPD patients, among which serum CYR61 level was the highest in patients with G 4. Pulmonary CYR61 was then detected in COPD patients and controls. As shown in Figure 1C, an obvious CYR61 staining was shown in the lungs from COPD patients. Quantitative analysis showed that pulmonary CYR61 was upregulated in COPD patients as compared with controls (Figure 1D). Moreover, pulmonary CYR61 was higher in G 3 and G 4 COPD patients than those in G 1-2 COPD patients (Figures 1E,F).

### Serum CYR61 Is Negatively Correlated With Lung Function Indexes in COPD Patients

**Figure 2:**
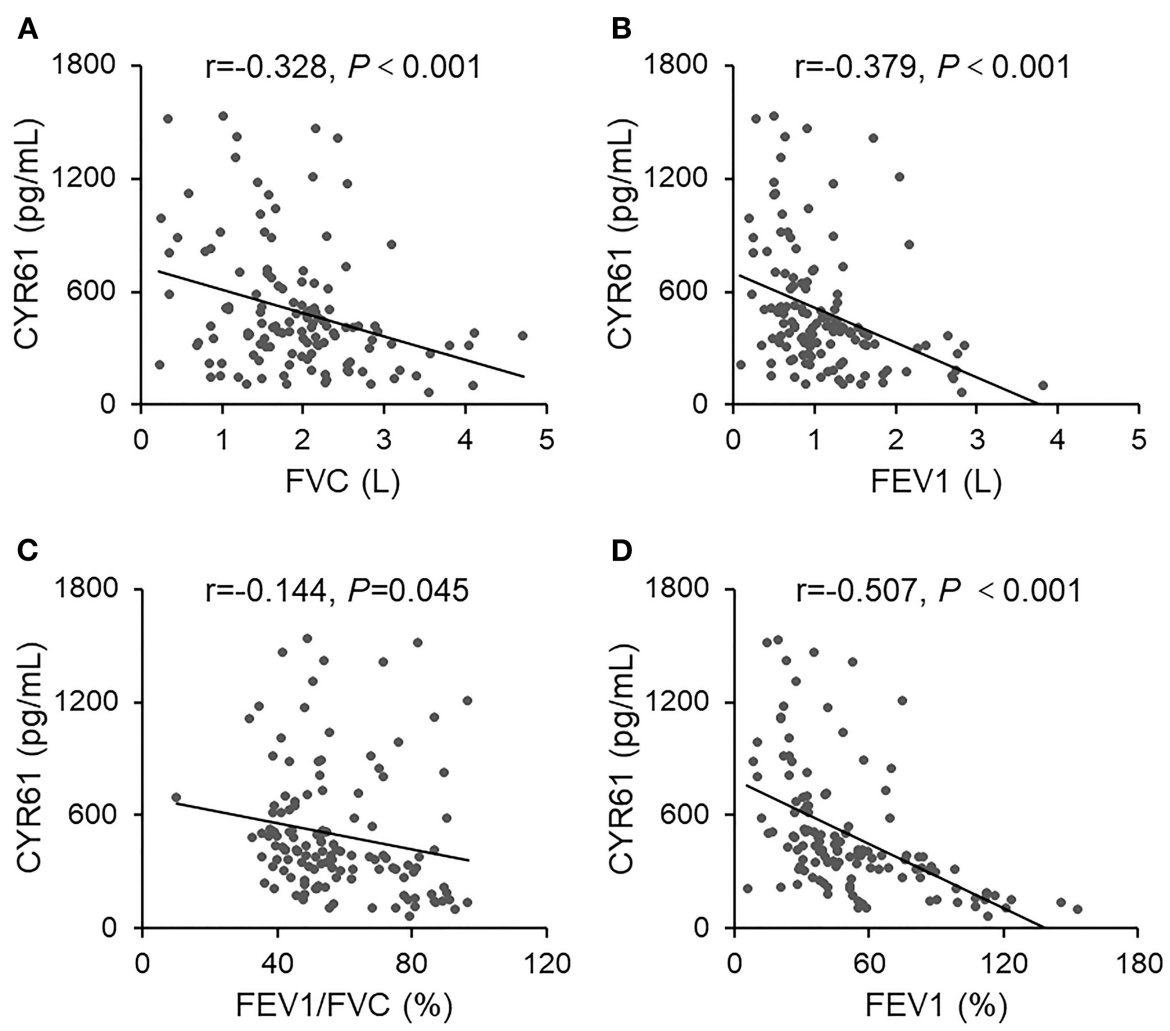
The correlation between serum CYR61 and lung function indexes in COPD patients. Pulmonary function was measured in 150 COPD patients. Sera were collected and CYR61 was detected using ELISA. Correlation between serum CYR61 and lung function indexes was analyzed. **(A)** CYR61 vs. FVC(L); **(B)** CYR61 vs. FEV1(L); **(C)** CYR61 vs. FEV1/FVC (%); **(D)** CYR61 vs. FEV1(%).

The correlation between serum CYR61 and FVC was analyzed among COPD patients. As expected, a negative correlation was observed between serum CYR61 and FVC (Figure 2A, r = −0.328, *P* < 0.001). The correlation between serum CYR61 and FEV1(L) was then analyzed among COPD patients. As shown in Figure 2B, there was a negative correlation between serum CYR61 and FEV1(L) (r = −0.379, *P* < 0.001). Next, the correlation between serum CYR61 and FEV1/FVC was evaluated among COPD patients. As shown in Figure 2C, there was a negative correlation between serum CYR61 and FEV1/FVC (r = −0.144, *P* = 0.045). The correlation between serum CYR61 and FEV1(%) is presented in Figure 2D. As expected, a negative correlation was observed between serum CYR61 and FEV1 (%) (r = −0.507, *P* < 0.001). Finally, regression analysis was used to evaluate the correlation between serum CYR61 and pulmonary function among COPD patients. Univariate regression analysis showed that there was a negative correlation between serum CYR61 and all lung function indexes in COPD patients (Table 2). Multivariate regression analysis was used to exclude the influence of age, gender and smoking on serum CYR61 level and pulmonary functions in COPD patients. Although no correlation between CYR61 and FEV1/FVC (%) was observed, there remains a negative correlation between serum CYR61 and other three lung function indexes in COPD patients (Table 2).

**Table 2:** Association of serum CYR61 with lung function.

**Variables**	Multivariable, β (95% CI)^*^	***P***
FEV1 (%)	−0.466 (−0.054, −0.028)	<0.001
FEV1/FVC (%)	−0.050 (−0.011, 0.006)	0.547
FEV1 (L)	−0.408 (−0.001, 0.000)	<0.001
FVC (L)	−0.406 (−0.001, 0.000)	<0.001

* *Age, gender, and smoking were adjusted*.

### An Increased CYR61 Is Associated With Pulmonary NF-κB Activation in COPD Patients

**Figure 3:**
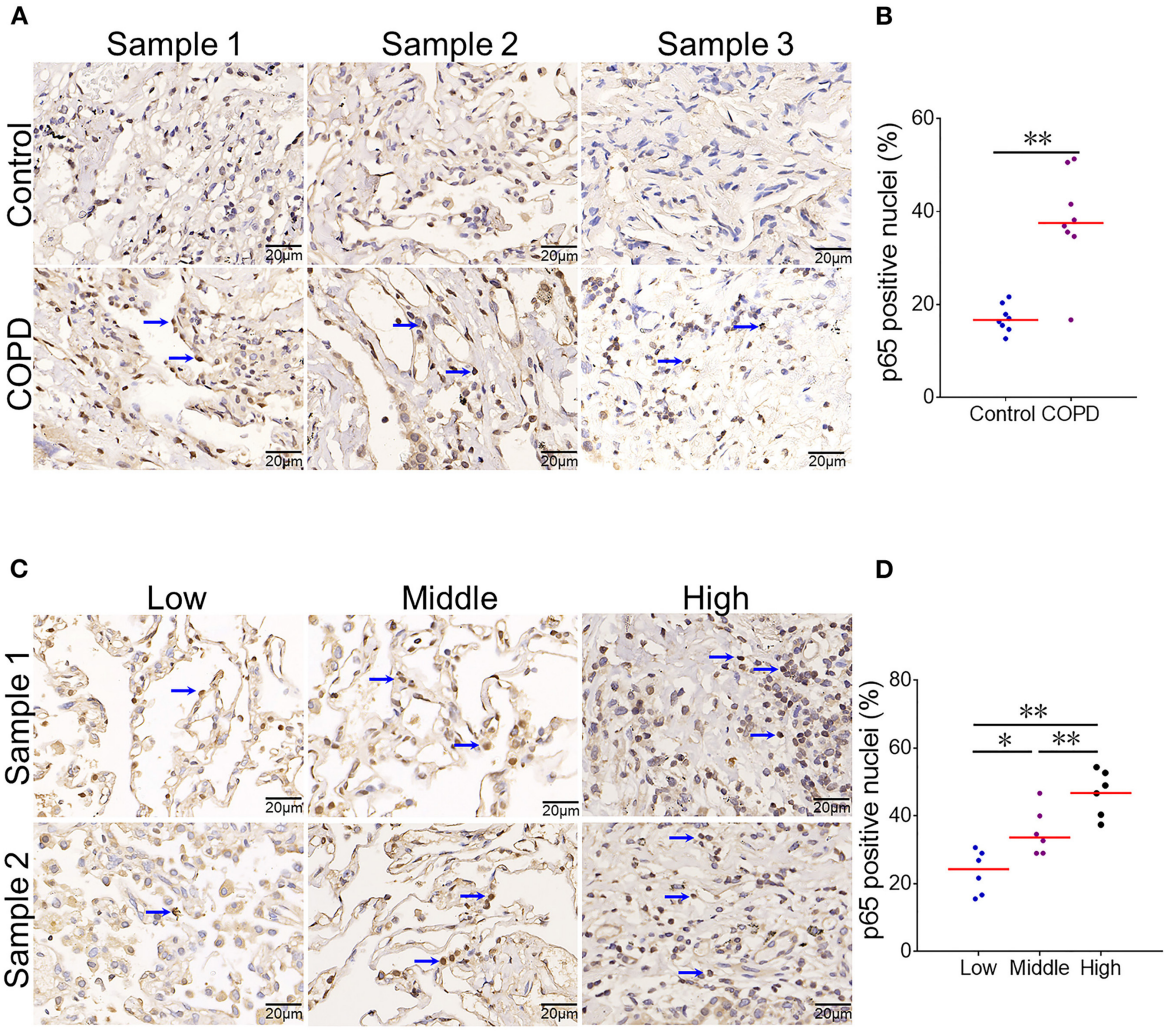
The association between serum CYR61 and pulmonary NF-κB activation in COPD patients. Lung tissues were collected from COPD patients and controls. Pulmonary NF-κB p65 was detected using IHC. **(A,B)** Pulmonary NF-κB p65-positive nuclei were compared between COPD patients and control subjects. **(A)** Three representative pictures: arrows indicate p65-positive nuclei; **(B)** Quantitative analysis of p65-positive nuclei in COPD patients and controls. **(C,D)** Pulmonary NF-κB p65-positive nuclei were compared among COPD patients with different levels of CYR61. **(C)** Three representative pictures: arrows indicate p65-positive nuclei; **(D)** Quantitative analysis of p65-positive nuclei in COPD patients with different levels of CYR61. All data were represented as means ± S.E.M. (*N* = 6). *P < 0.05, **P < 0.01.

Pulmonary NF-κB was evaluated among COPD patients and controls. As expected, numerous NF-κB p65-positive nuclei, as determined by IHC, were observed in the lung of COPD patients (Figure 3A). Quantitative analysis showed that NF-κB p65-positive nuclei were elevated in COPD patients as compared with controls (Figure 3B). The association between serum CYR61 and pulmonary NF-κB activation was analyzed in COPD patients. As expected, the numbers of pulmonary NF-κB p65-positive nuclei were more in COPD patients with high level of CYR61 than in COPD patients with low level of CYR61 (Figures 3C,D).

### An Increased CYR61 Is Associated With Serum MCP-1 and TNF-α in COPD Patients

**Figure 4:**
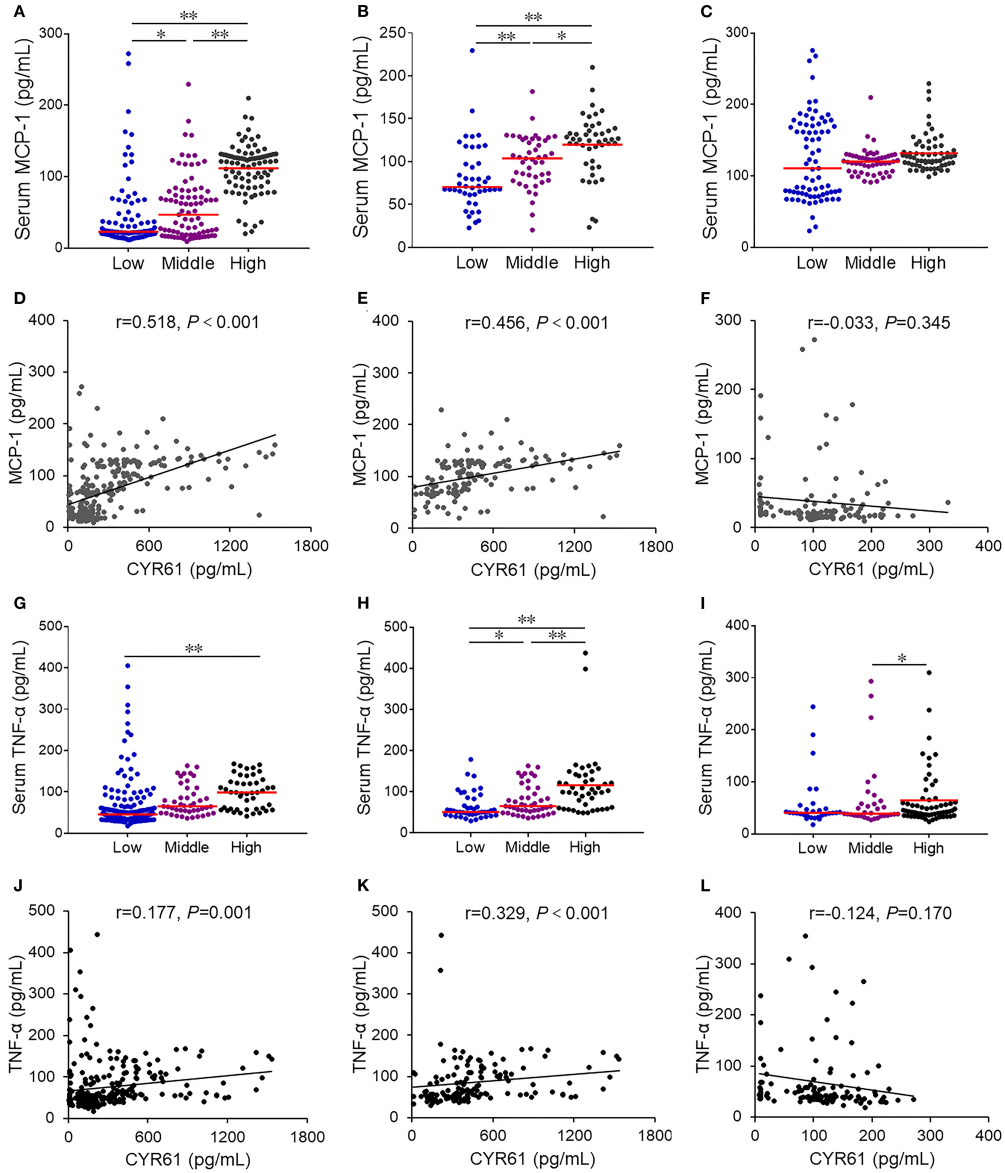
The association of serum CYR61 level with serum inflammatory cytokines in COPD patients. Sera were collected from 150 COPD patients and 150 control subjects. Serum CYR61 and inflammatory cytokines were detected using ELISA. **(A,D)** Association between serum CYR61 and MCP-1 was analyzed among all subjects. **(A)** Serum MCP-1 was compared among subjects with different levels of CYR61. **(D)** Correlation analysis between serum CYR61 and MCP-1. **(B,E)** Association between serum CYR61 and MCP-1 was analyzed among COPD patients. **(B)** Serum MCP-1 was compared among COPD patients with different levels of CYR61. **(E)** Correlation analysis between serum CYR61 and MCP-1. **(C,F)** Association between serum CYR61 and MCP-1 was analyzed among control subjects. **(C)** Serum MCP-1 was compared among control subjects with different levels of CYR61. **(F)** Correlation analysis between serum CYR61 and MCP-1. **(G,J)** Association between serum CYR61 and TNF-α was analyzed among all subjects. **(G)** Serum TNF-α was compared among subjects with different levels of CYR61. **(J)** Correlation analysis between serum CYR61 and TNF-α. **(H,K)** Association between serum CYR61 and TNF-α was analyzed among COPD patients. **(H)** Serum TNF-α was compared among COPD patients with different levels of CYR61. **(K)** Correlation analysis between serum CYR61 and TNF-α. **(I,L)** Association between serum CYR61 and TNF-α was analyzed among control subjects. **(I)** Serum TNF-α was compared among control subjects with different levels of CYR61. **(L)** Correlation analysis between serum CYR61 and TNF-α. *P < 0.05, **P < 0.01.

Firstly, the associations between serum CYR61 and inflammatory cytokines was analyzed among all subjects. As expected, serum MCP-1 was gradually elevated in parallel with CYR61, among which serum MCP-1 level was the highest in subjects with high level of CYR61 (Figure 4A). Correlation analysis showed a positive association between serum CYR61 and MCP-1 (Figure 4D, r = 0.518, *P* < 0.001). A positive correlation, as determined by multivariate regression analysis that excluded the influence of age, gender and smoking, was observed between serum CYR61 and MCP-1 in all subjects (Table 3). Next, the association between serum CYR61 and MCP-1 was analyzed among COPD patients. As expected, serum MCP-1 was gradually elevated in parallel with CYR61, among which serum MCP-1 level was the highest in subjects with high level of CYR61 (Figure 4B). Correlation analysis showed a positive association between serum CYR61 and MCP-1 (Figure 4E, r = 0.456, *P* < 0.001). Moreover, a positive correlation, as determined by multivariate regression analysis that excluded the influence of age, gender and smoking, was observed between serum CYR61 and MCP-1 in COPD patients (Table 3). Finally, the association between serum CYR61 and MCP-1 was analyzed among control subjects. As shown in Figure 4C, no significant difference on serum MCP-1 was observed among different groups. Correlation and multivariate regression analyses showed that there was no association on serum CYR61 and MCP-1 (Figure 4F; Table 3, *P* > 0.05). Moreover, the association of serum CYR61 and TNF-α was evaluated in all cases. As shown in Figure 4G, serum TNF-α was increased in high serum CYR61 group than these in low serum CYR61 group. Besides, serum TNF-α was gradually risen in line with serum CYR61 in COPD patients (Figure 4H). In addition, serum TNF-α was elevated in High group than in Middle group among control cases (Figure 4I). Although, there was no obvious correlation of serum TNF-α with CYR61 in control cases (Figure 4L), serum CYR61 was positively associated with TNF-α in all cases (Figure 4J, r = 0.177, *P* = 0.001) and COPD patients (Figure 4K, r = 0.329, *P* < 0.001).

**Table 3:** Associations of serum CYR61 with MCP-1 and TNF-α.

**Groups**	**MCP-1**		**TNF-α**	
	**Multivariable,β (95% CI)**	***P***	**Multivariable,β (95% CI)**	***P***
All cases	0.479 (0.067, 0.102)	<0.001	0.177 (0.316, 1.685)	<0.001
Control	−0.027 (−0.145, 0.104)	0.747	−0.156 (−0.316, 0.020)	0.083
COPD	0.473 (0.041,0.077)	<0.001	0.327 (1.467, 4.365)	<0.001

*Age, gender, and smoking were adjusted*.

### The Mediating Effect of Inflammatory Cytokines Between Increased CYR61 and Decreased Lung Function Indexes in COPD Patients

**Figure 5:**
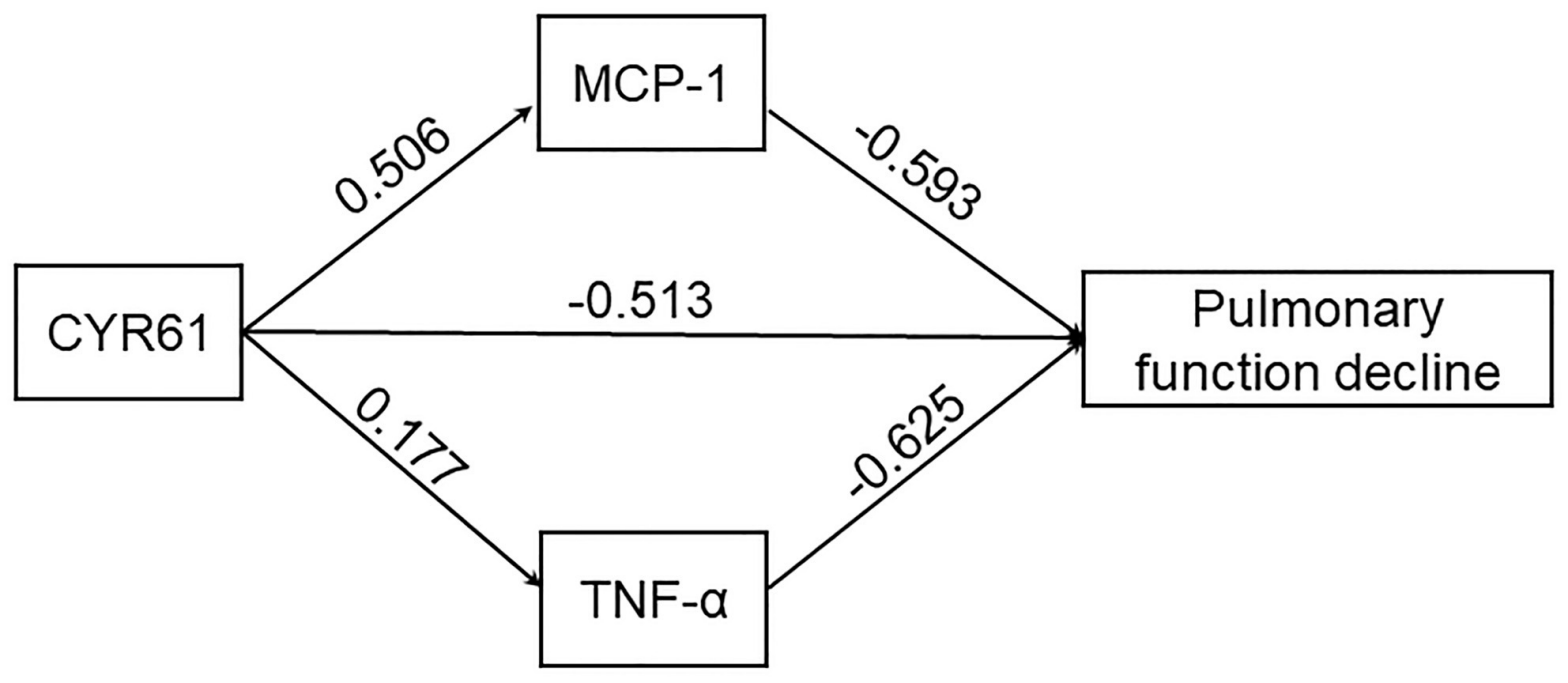
Postulated mediational path model.

First, the direct effect of increased CYR61 on pulmonary function decline was analyzed. As shown in Figure 5, serum CYR61 was negatively associated with FEV1 (%) (β = −0.513, *P* < 0.01) in COPD patients. The mediating effect of inflammatory cytokines between CYR61 and lung function indexes was then evaluated in COPD patients. As shown in Figure 5, obvious mediating effects between increased MCP-1 (β = −0.300, *P* < 0.05) and TNF-α (β = −0.111, *P* < 0.05) with decreased lung function were observed in COPD patients. The total effect of CYR61 on lung function decline was −0.924 (*P* < 0.01) in COPD patients. The relative contribution of MCP-1 and TNF-α in CYR61 elevation-induced pulmonary function decline was 44.5% in COPD patients.

### An Increased Serum CYR61 Is Positively Associated With Hospital Stays in COPD Patients

According to quartile, serum CYR61 was classified as Low group (<410.0 pg/ml) and High group (≥410.0 pg/ml), hospital stays were divided into Short group (<10 days) and Long group (≥10 days). Then, the effect of serum CYR61 at the early stage on hospital stay was evaluated in Table 4. Among 150 COPD patients, the hospital stays of 69 (46.0%) patients was longer than 10 days. The number of cases with higher hospital stays was more among COPD patients with high serum CYR61 than those with low serum CYR61 (55.1 vs. 25.9%; RR = 2.124, 95% Cl: 1.388~3.250; *P* < 0.001).

**Table 4:** Association of serum CYR61 and hospital stays in COPD patients.

**CYR61**	**Cases, *n***	**Longer hospital stays, *n* (%)**	**RR (95%)**	***P***
Low	81	21 (25.9)	1	-
High	69	38 (55.1)	2.124 (1.388, 3.250)	<0.001

*According to quartile, serum CYR61 was classified as Low group (<410.0 pg/ml) and High group (≥410.0 pg/ml), hospital stays were divided into Short group (<10 days) and Long group (≥10 days)*.

*RR, relative risk*.

## Discussion

The associations of serum CYR61 with pulmonary inflammation and lung function indexes were investigated in COPD patients. The major findings were as follow: Firstly, serum CYR61 level was elevated and pulmonary CYR61 expression was upregulated in COPD patients; Secondly, an increased CYR61 was associated with decreased lung function indexes in COPD patients; Thirdly, an increased CYR61 is associated with an elevation of serum MCP-1, TNF-α and activation of pulmonary inflammatory signaling in COPD patients. Fourthly, an increased CYR61 is associated with a longer hospital stays among COPD patients. Our results suggest that pro-inflammatory cytokine and chemokine are mediators between CYR61 elevation and COPD progression.

Accumulating data indicate that CYR61 is involved in the pathogenesis of COPD (18). An early study found that pulmonary CYR61 was upregulated in COPD patients as compared with controls (22). Indeed, cigarette smoking is a major risk factor for COPD (27). According to an earlier report, cigarette smoke extracts upregulated CCN1 through evoking excess reactive oxygen spices and endoplasmic reticulum stress (28). In the current study, we measured serum CYR61 level and pulmonary CYR61 expression in COPD patients. Our results showed that serum CYR61 was elevated and pulmonary CYR61 was upregulated in COPD patients. Interestingly, we found that serum CYR61 level and pulmonary CYR61 expression were positively associated with the severity of COPD. To further determine the role of CYR61 in COPD progression, the current study analyzed the association between serum CYR61 level and lung function indexes in COPD patients. We found that serum CYR61 level is negatively correlated with several lung function indexes in COPD patients. Not only that, the association of serum CYR61 and the prognosis was estimated in COPD patients. Our results indicated that serum higher CYR61 on admission prolonged the hospital stays among COPD patients. Our results provide evidence for the first time that CYR61 may be a biomarker to predict COPD progression.

The mechanism by which CYR61 associates COPD progression remains unclear. Numerous studies demonstrated that chronic airway inflammation aggravated lung function decline in COPD patients (5, 29). On the other hand, several studies found that CYR61 had proinflammatory activities (30–34). An earlier study showed that CYR61 mediated cigarette smoke extracts evoked IL-8 secretion by lung epithelial cells (28). The current study investigated the association of serum CYR61 with serum inflammatory cytokines and inflammatory signaling in the lungs of COPD patients. Our results showed that pulmonary NF-κB p65-positive nuclei were higher in COPD patients with high CYR61 than with low CYR61. Accordingly, serum MCP-1 and TNF-α, the downstream pro-inflammatory cytokine and chemokine of NF-κB signaling, were increased in parallel with CYR61, among which serum inflammatory cytokines were the highest in COPD patients with high CYR61. To determine the mediating effect of inflammatory cytokines between increased CYR61 and decreased lung function indexes, we analyzed the link between serum CYR61 and inflammatory cytokines in COPD patients. Despite no association between serum CYR61 and inflammatory cytokines among control subjects, a positive correlation of serum CYR61 with TNF-α and MCP-1, as determined by multivariate regression analysis that excluded the influence of age, gender and smoking, was observed among COPD patients. Further analysis showed an obvious mediating effect between an increased inflammatory cytokines with a decreased lung function indexes in COPD patients.

There are several flaws in the current study. First, the results of the current study were from a cross-sectional analysis, in which all COPD patients were from Anhui COPD Cohort. The causal link among CYR61, pulmonary inflammation and COPD progression was not clear. Thus, further follow-up observation and animal experiments are needed to determine the influence of CYR61 on pulmonary inflammation and lung function indexes in COPD patients. Second, the current study has not clarified the underlying mechanism through which CYR61 upregulates inflammatory cytokines in COPD patients. Indeed, an earlier study found that CYR61 upregulated MCP-1 through activating NF-κB pathway in vascular endothelial cells (33). In addition, the previous study has revealed that CYR61 elevated TNF-α via NF-κB signal in murine macrophages (30). Additional experiment is required to explore the exact mechanism by which CYR61 activates NF-κB in the lungs of COPD patients. Third, CYR61 was only detected in lung tissues and sera. But, the level of CYR61 was unclear in bronchoalveolar lavage of COPD patients. In addition, cell localization of CYR61 was not conducted in COPD patients. Further experiments are required to resolve this puzzle in the future work.

## Conclusion

In summary, the current study investigated the association among serum CYR61, pulmonary inflammation and lung function indexes among COPD patients. Our results showed that serum CYR61 and MCP-1 were elevated in COPD patients. We found that an increased CYR61 was correlated with decreased lung function indexes in COPD patients. Moreover, an increased CYR61 was associated with pulmonary NF-κB activation and serum MCP-1 increase in COPD patients. In addition, an increased CYR61 is associated with a longer hospital stays among COPD patients. Our results provide evidence that CYR61 can be used as a biomarker to predict COPD progression. Inflammatory chemokine MCP-1 may be a mediator between CYR61 and lung function decline in COPD patients.

## Data Availability Statement

The raw data supporting the conclusions of this article will be made available by the authors, without undue reservation.

## Ethics Statement

The current study was approved by the Ethics Committee of Anhui Medical University (2021030). The patients/participants provided their written informed consent to participate in this study.

## Author Contributions

D-XX, HW, Z-XT, and LF contributed to the study design, analyzing data, and preparation manuscript. W-JW, PZ, and HZ were involved in the acquisition of data. D-XX and LF worked on the study concept, design, and final proof. All authors have read and approved the final manuscript.

## Funding

This project was supported by National Natural Science Foundation of China (81800062).

## Conflict of Interest

The authors declare that the research was conducted in the absence of any commercial or financial relationships that could be construed as a potential conflict of interest.

## Publisher's Note

All claims expressed in this article are solely those of the authors and do not necessarily represent those of their affiliated organizations, or those of the publisher, the editors and the reviewers. Any product that may be evaluated in this article, or claim that may be made by its manufacturer, is not guaranteed or endorsed by the publisher.

## Abbreviations

COPD: Chronic obstructive pulmonary disease
CYR61: Cysteine-rich 61
NF-κB: nuclear factor-kappa B
MCP-1: Macrophage chemoattractant protein-1
FEV1: Forced expiratory volume in 1 s
FVC: forced vital capacity.
