# Corrigendum: Serum CYR61 is associated with airway inflammation and is a potential biomarker for severity in chronic obstructive pulmonary disease

**DOI:** 10.3389/fmed.2023.1236279

**Published:** 2023-07-21

**Authors:** Zhu-Xia Tan, Lin Fu, Wen-Jing Wang, Ping Zhan, Hui Zhao, Hua Wang, De-Xiang Xu

**Affiliations:** ^1^Department of Toxicology, Anhui Medical University, Hefei, China; ^2^Second Affiliated Hospital, Anhui Medical University, Hefei, China

**Keywords:** CYR61, COPD, NF-κB, lung function, inflammatory cytokines

In the published article, there was an error in affiliation. The affiliations were listed in the incorrect order. They should appear as:

^1^ Department of Toxicology, Anhui Medical University, Hefei, China

^2^ Second Affiliated Hospital, Anhui Medical University, Hefei, China

The authors apologize for this error and state that this does not change the scientific conclusions of the article in any way. The original article has been updated.

