# Supplementary material for: Serum CYR61 Is Associated With Airway Inflammation and Is a Potential Biomarker for Severity in Chronic Obstructive Pulmonary Disease
Source: Front Med (Lausanne). 2021 Nov 30;8:781596. doi: 10.3389/fmed.2021.781596 (PMC8669148; doi:10.3389/fmed.2021.781596)
Supplement: Supplementary file 1 [file Table_1.doc]

Supplemental Table 1. Demographic information and clinical characteristics in COPD patients

| Variable | G 1-2 (n=64) | G 3 (n=51) | G 4 (n=35) | *P* |
| --- | --- | --- | --- | --- |
| Emphysema, n (%) | 47 (73.4) | 44 (54.5) | 29 (82.9) | 0.206 |
| Pulmonary function |  |  |  |  |
| FEV1 (%) | 79.37±3.437 | 37.92±0.736** | 21.16±1.251**^##^ | ＜0.001 |
| FEV1/FVC (%) | 68.99±2.083 | 47.94±1.902** | 51.28±2.860** | ＜0.001 |
| FEV1 (L) | 1.61±0.090 | 0.89±0.033** | 0.50±0.034**^##^ | ＜0.001 |
| FVC (L) | 2.38±0.117 | 1.87±0.074** | 1.07±0.095**^##^ | ＜0.001 |
| RV%TLC-SB (%) | 50.18±2.215 | 58.38±2.020* | 63.58±3.508** | 0.004 |
| DLCO SB (mmol/min/kPa) | 4.72±0.368 | 2.75±0.256** | 2.40±0.232** | ＜0.001 |
| Blood gas |  |  |  |  |
| PCO2 (mmHg) | 50.76±3.813 | 55.58±12.884 | 56.41±4.430 | 0.520 |
| PO2 (mmHg) | 76.75±4.023 | 63.44±2.900 | 72.96±8.151 | 0.087 |
| PH | 7.38±0.012 | 7.39±0.010 | 7.40±0.015 | 0.649 |

Compared with G 1-2, **P*<0.05, ***P*<0.01.

Compared with G 3, ^##^*P*<0.01.
