# Supplementary figures and images for: Serum CYR61 Is Associated With Airway Inflammation and Is a Potential Biomarker for Severity in Chronic Obstructive Pulmonary Disease
Source: Front Med (Lausanne). 2021 Nov 30;8:781596. doi: 10.3389/fmed.2021.781596 (PMC8669148; doi:10.3389/fmed.2021.781596)

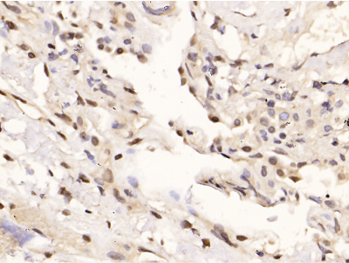

Supplement: Supplementary file 2 [file Data_Sheet_1.ZIP › CYR61/COPD/1.tif]

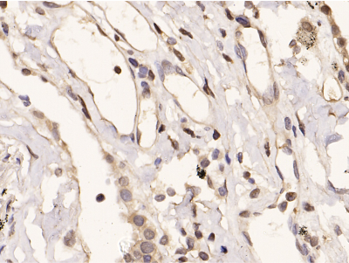

Supplement: Supplementary file 2 [file Data_Sheet_1.ZIP › CYR61/COPD/2.tif]

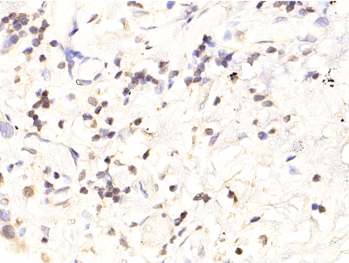

Supplement: Supplementary file 2 [file Data_Sheet_1.ZIP › CYR61/COPD/3.tif]

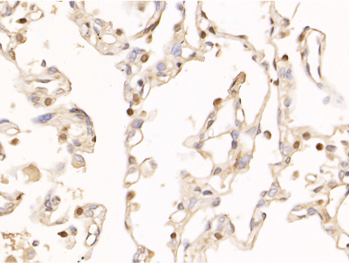

Supplement: Supplementary file 2 [file Data_Sheet_1.ZIP › CYR61/COPD/4.tif]

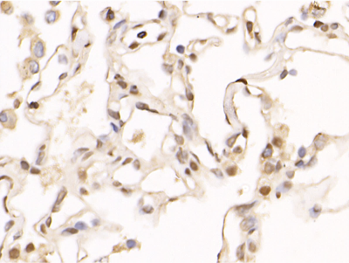

Supplement: Supplementary file 2 [file Data_Sheet_1.ZIP › CYR61/COPD/5.tif]

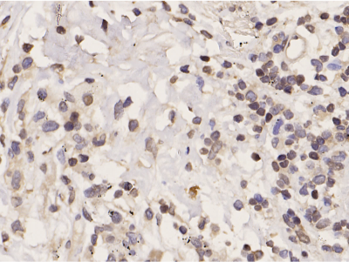

Supplement: Supplementary file 2 [file Data_Sheet_1.ZIP › CYR61/COPD/6.tif]

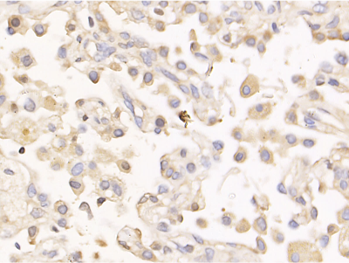

Supplement: Supplementary file 2 [file Data_Sheet_1.ZIP › CYR61/COPD/7.tif]

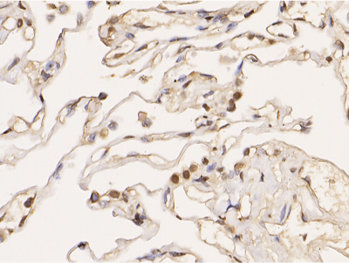

Supplement: Supplementary file 2 [file Data_Sheet_1.ZIP › CYR61/COPD/8.tif]

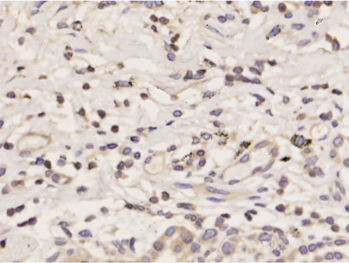

Supplement: Supplementary file 2 [file Data_Sheet_1.ZIP › CYR61/COPD/9.tif]

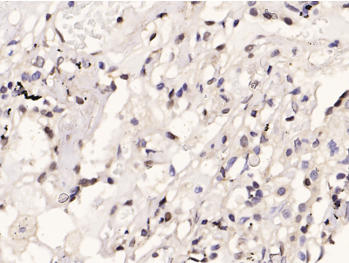

Supplement: Supplementary file 2 [file Data_Sheet_1.ZIP › CYR61/Control/1.tif]

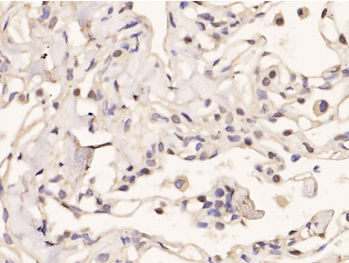

Supplement: Supplementary file 2 [file Data_Sheet_1.ZIP › CYR61/Control/2.tif]

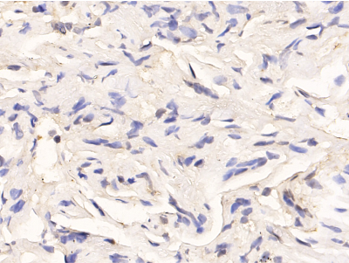

Supplement: Supplementary file 2 [file Data_Sheet_1.ZIP › CYR61/Control/3.tif]

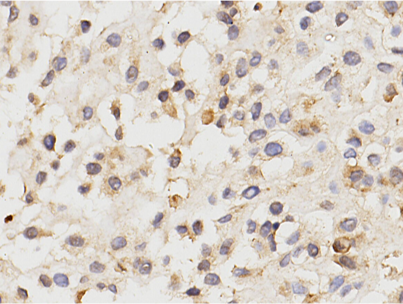

Supplement: Supplementary file 3 [file Data_Sheet_2.ZIP › NF-a╩B/COPD/1.tif]

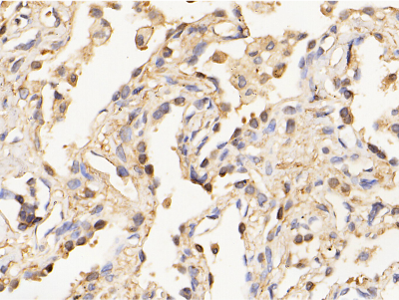

Supplement: Supplementary file 3 [file Data_Sheet_2.ZIP › NF-a╩B/COPD/2.tif]

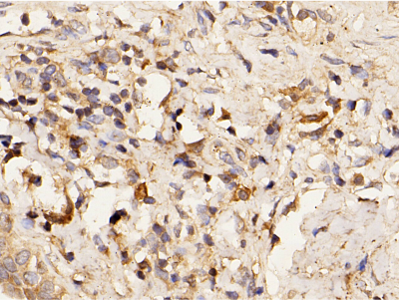

Supplement: Supplementary file 3 [file Data_Sheet_2.ZIP › NF-a╩B/COPD/3.tif]

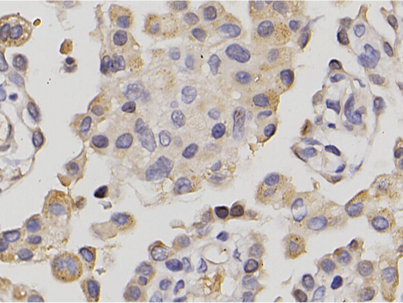

Supplement: Supplementary file 3 [file Data_Sheet_2.ZIP › NF-a╩B/COPD/4.tif]

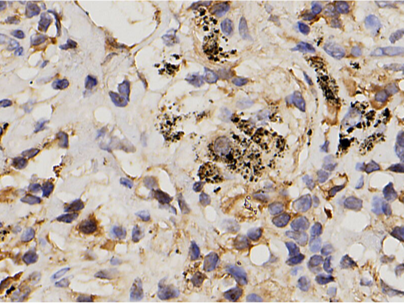

Supplement: Supplementary file 3 [file Data_Sheet_2.ZIP › NF-a╩B/COPD/5.tif]

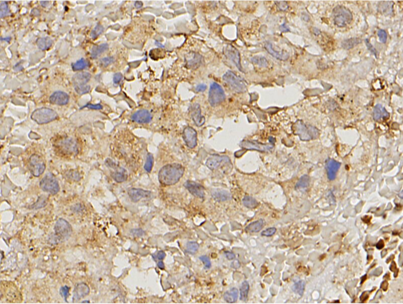

Supplement: Supplementary file 3 [file Data_Sheet_2.ZIP › NF-a╩B/COPD/6.tif]

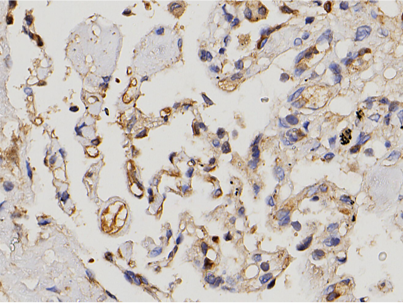

Supplement: Supplementary file 3 [file Data_Sheet_2.ZIP › NF-a╩B/COPD/7.tif]

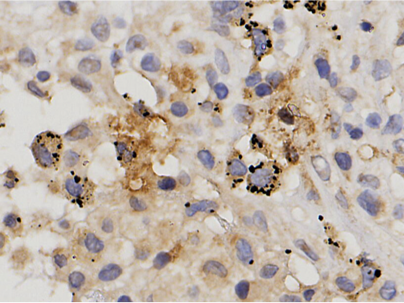

Supplement: Supplementary file 3 [file Data_Sheet_2.ZIP › NF-a╩B/COPD/8.tif]

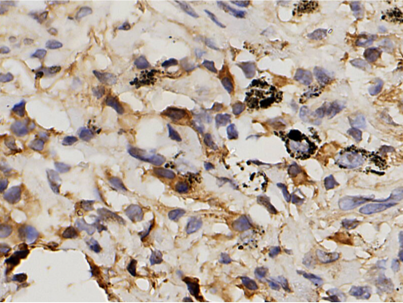

Supplement: Supplementary file 3 [file Data_Sheet_2.ZIP › NF-a╩B/COPD/9.tif]

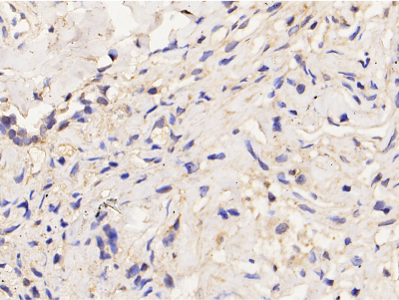

Supplement: Supplementary file 3 [file Data_Sheet_2.ZIP › NF-a╩B/Control/1.tif]

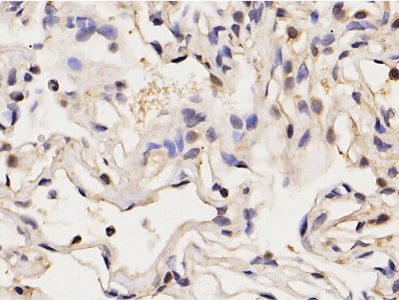

Supplement: Supplementary file 3 [file Data_Sheet_2.ZIP › NF-a╩B/Control/2.tif]

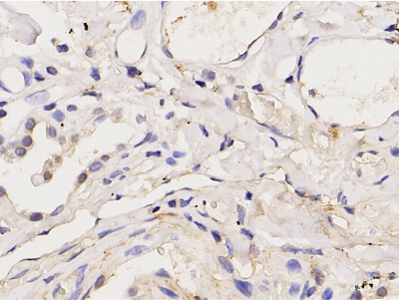

Supplement: Supplementary file 3 [file Data_Sheet_2.ZIP › NF-a╩B/Control/3.tif]
